# Signal increase for dipiridamol induced stress on cardiac magnetic resonance perfusion in minipig

**DOI:** 10.1186/1532-429X-16-S1-P75

**Published:** 2014-01-16

**Authors:** Bernd M Muller-Bierl, Kaoru Tanaka, Yves Fierens, Nico Buls, Toon van Cauteren, Inneke Willekens, Sigrid van Laere, Johan de Mey

**Affiliations:** 1 Radiologie, Universitair Ziekenhuis, Jette-Brussels, Belgium

## Background

We compare eight reported methods for the analysis of cardiac perfusion flow from DCE data with respect to their potential in measuring a signal increase in the heart tissue by application of medication induced stress. Our work will strongly concern cardiologists, radiologists and clinical scientists performing myocardial perfusion imaging at high field (3 T).

## Methods

A healthy minipig, measured 5 times using first pass DCE imaging. We sampled the arterial input function (AIF) and the tissue response function (TRF), from the mid-ventricular tissue region from anterior to inferior in the short axis view of the heart, using incoherent imaging (Steady State Incoherent, SSI, or FLASH), and coherent imaging (Steady State Coherent, SSC, or BALANCED) [1] twice, without and with dipiridamole induced stress. We compared the results from analysis methods taken from the literature, namely the Fermi function (1, 'Fermi'), Finite Differences (2, 'FD'), 2 Compartment Tofts (3, '2CTM'), Exchange (4, '2CXM'), Uptake (5,'2CUM'), Tofts (6,'1CTM'), Patlak (7,'Patlak'), and the Maximum Slope (8, 'upslope') method [2]. We calculated the correlation between the flow computed using the different methods and a selected, arithmetic mean of methods 1-3, 6, for non-stress and stress induced AIFs and TRFs, We corrected for the different physiological pathways to heart tissue from the vascular system by division through a ratio ATRF/AAIF, to keep the actual ratio constant for all 5 measurements. The first 'A' therefore designates the area under the measured signal. We discarded data that was considered to be non-physiological (only flow obeying 10 < PF < 200 ml/100 ml/min was accepted for our statistical analysis).

## Results

**Figure 1:**
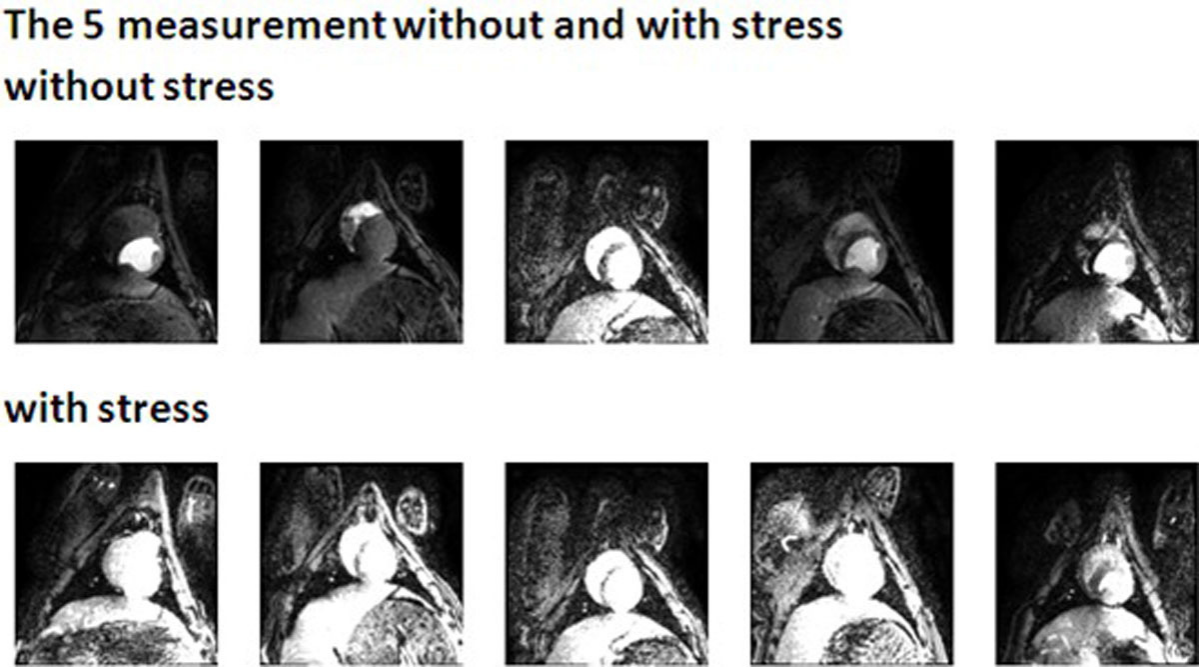
**Effect of the signal enhancement during contrast inflow by dipiridamole-induced stress**.

**Figure 2:**
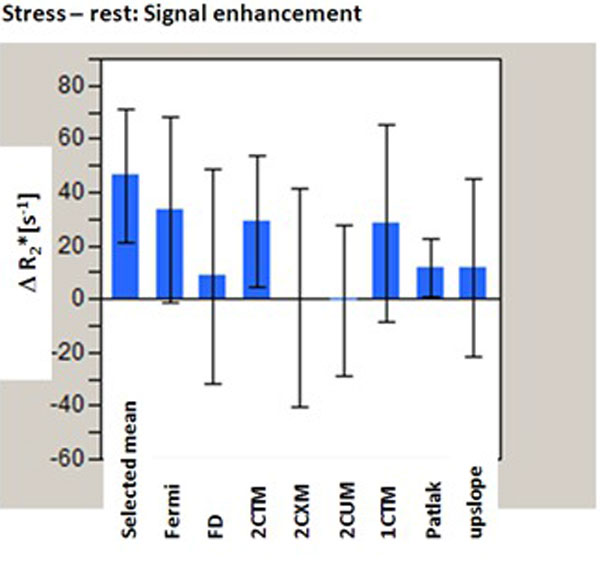
**Comparison of absolute signal enhancement between perfusion flow from the various methods and from the selected, arithmetic mean**.

Signal enhancement by the passage of the prebolus and the bolus of contrast agent was apparently different in the 5 consecutive measurements (Figure 1). The selected mean 1-3, 6, was very sensitive to stress induction in the heart. It was even better than either of the single method estimates (Figure 2).

## Conclusions

We corrected for the different tissue pathways from the venous system to the tissue region in the heart. Measurement of perfusion enhancement during medication induced stress then can best be done by taking an appropriate selected, arithetic mean of analysis methods. Image correction should be tried out in aim to get better quality of the AIF and TRF used for the perfusion flow estimate. Figure 1: Effect of the signal enhancement during contrast inflow by dipiridamole-induced stress. Figure 2: Comparison of absolute signal enhancement between perfusion flow from the various methods and from the selected, arithmetic mean.

## Funding

Internal (hospital) funding.

